# Case report: Emerging BRCA mutation confers benefit from olaparib after chemotherapy intolerance in advanced triple‐negative breast cancer

**DOI:** 10.1002/ccr3.8680

**Published:** 2024-04-03

**Authors:** Xia‐Bo Shen, Jia‐Yi Wu, Jia‐ying Li, Xi‐Ying Shao, Xiao‐Jia Wang

**Affiliations:** ^1^ Department of Breast Medical Oncology Cancer Hospital of the University of Chinese Academy of Sciences (Zhejiang Cancer Hospital) Hangzhou China; ^2^ Wenzhou Medical University Wenzhou China

**Keywords:** BRCA mutation, case report, olaparib, triple‐negative breast cancer

## Abstract

**Key Clinical Message:**

In a patient with metastatic breast cancer, an acquired BRCA mutation in the BRCA gene was detected, resulting in benefits from olaparib treatment. This underscores the importance of ongoing genetic phenotype testing after paclitaxel chemotherapy.

**Abstract:**

Triple‐negative breast cancer (TNBC) is associated with a poor prognosis and elevated mortality risk. BRCA mutations are commonly regarded as prevalent mutations in TNBC patients, strongly associated with congenital familial heredity. Dynamic changes in mutation sites, however, are rarely reported. In this case report, we report a 59‐year‐old TNBC patient who developed pulmonary metastases post‐chemoradiotherapy. No BRCA mutations were detected through NGS. After 7.6 months of nab‐paclitaxel treatment, the patient experienced progression of lung metastases, and BRCA mutations were detected through NGS testing. Subsequent administration of olaparib resulted in a reduction in lung metastasis, demonstrating significant therapeutic efficacy. This case underscores the infrequent occurrence of treatment‐induced BRCA mutations and emphasizes the significance of dynamic NGS genetic testing for real‐time assessment of a patient's mutational status.

## 1 INTRODUCTION

Olaparib is an oral poly (adenosine diphosphate–ribose) polymerase inhibitor, and it has antitumor activity in metastatic breast cancer patients with germline BRCA mutation.^1^, ^2^ Preventive and surveillance strategies for BRCA pathogenic variant breast cancer can reduce cancer recurrence and improve treatment outcomes.^3^ This article reported an advanced triple‐negative breast cancer patient with an emerging BRCA mutation, and she benefited from olaparib after chemotherapy intolerance. The BRCA mutation is considered a classic familial hereditary gene, and we have observed dynamic changes in the patient's genetic phenotype during the progression of the tumor. Throughout the patient's extended antitumor treatment, factors such as chemotherapy drugs may induce alterations in the tumor gene phenotype, thereby impacting the patient's survival. Therefore, it is essential to conduct long‐term monitoring of the patient's tumor gene phenotype.

## 2 CASE HISTORY/EXAMINATION/PRESENTATION

### 2.1 Case presentation

The patient, a 59‐year‐old postmenopausal woman, sought medical attention at our hospital in July 2021 following the discovery of multiple lung metastases after undergoing breast cancer surgery. In 2018, she visited a local hospital due to a lump detected during her annual physical examination. An ultrasound examination revealed a mass in her right breast, measuring approximately 3.3 × 2.4 × 2.1 cm. On October 19, 2018, a core needle biopsy of the right breast mass was performed, and the pathological report indicated invasive ductal carcinoma, grade III. Further immunohistochemistry (IHC) analysis revealed ER (1+, <1%), PR (−), HER‐2 (−), and Ki‐67 (60%–70%). Subsequently, the patient underwent neoadjuvant therapy with the EC regimen (EPI‐ADM 145 mg on Day 1 + CTX 0.95 g on Day 1, q3w) followed by four cycles of the T regimen (docetaxel 150 mg on Day 1, q3w).

**FIGURE 1:**
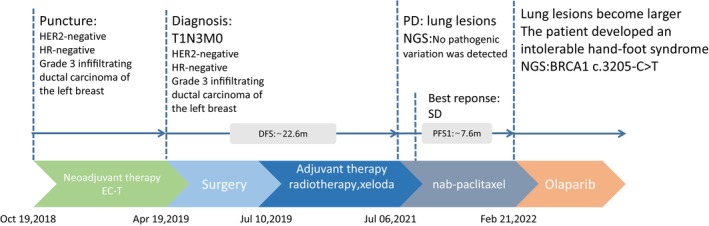
Patient management. EC‐T: EC sequence T regimen: four‐cycle EC regimen (EPI‐ADM + CTX 0.95g d1, q3w) sequence four‐cycle T regimen (docetaxel).; NGS: next‐generation sequencing; SD: stable disease; PD: progressive disease.

On April 19, 2019, the patient underwent right breast‐conserving surgery, involving local extended excision, axillary lymph node dissection, and free glandular flap reconstruction. The postoperative pathological report revealed right breast invasive ductal carcinoma, grade III, measuring about 1.5 × 1.2 × 1.2 cm, with a Miller–Payne grade II and negative resection margins. Seven out of 16 axillary lymph nodes were involved. The IHC report showed ER (−), PR (−), HER‐2 (−), and Ki‐67 (+, 60%–70%). Postoperative adjuvant radiotherapy was administered to the right breast, right clavicle area, and internal mammary lymph nodes, with a dose of DT 50 Gy/25F, 2.0 Gy/F, 5F/W. Additionally, the patient received eight cycles of oral capecitabine (2.0 g in the morning and 1.5 g in the evening, Days 1–14) after surgery (Figure 1).

**FIGURE 2:**
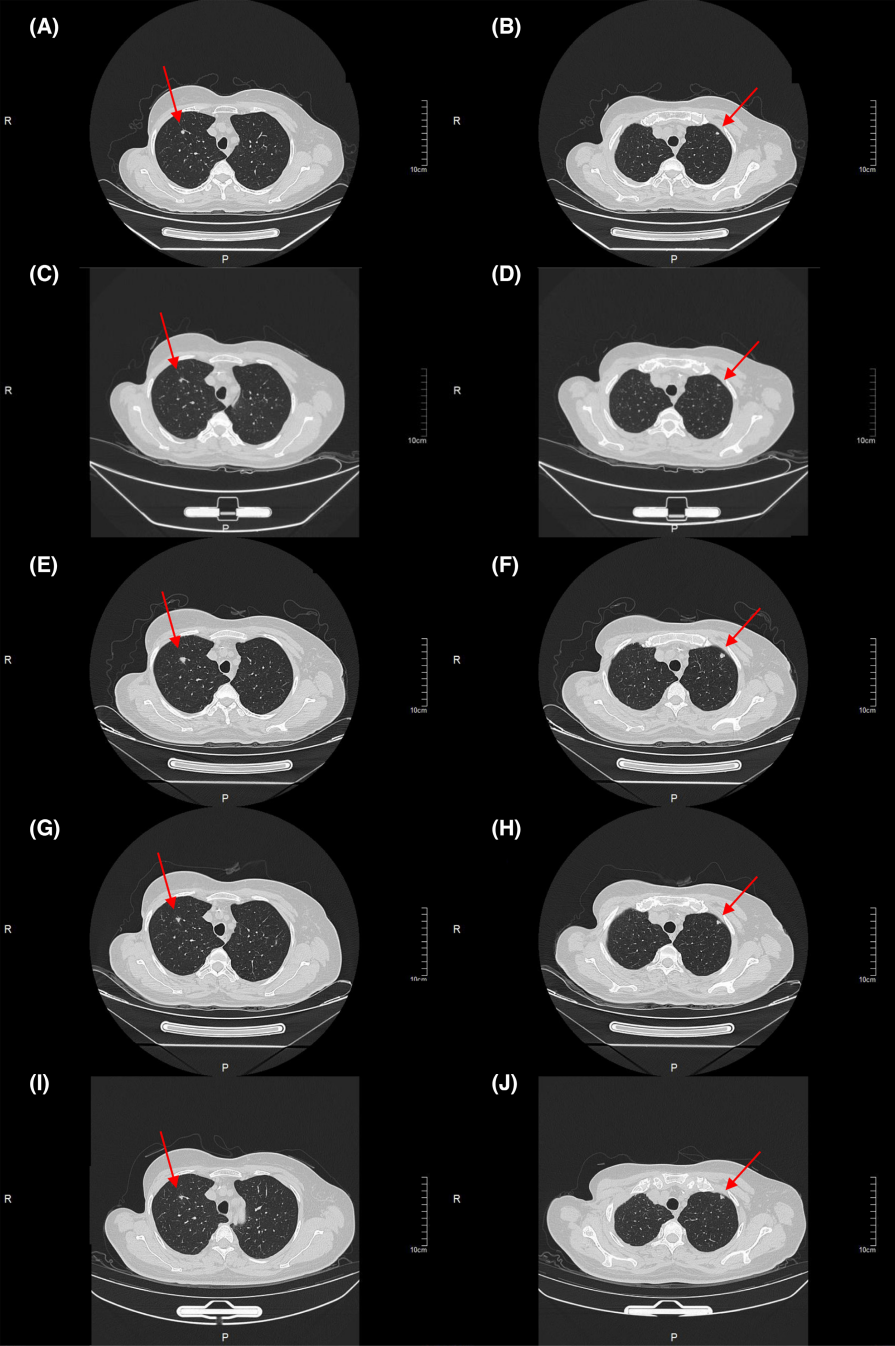
CT Image scans for lung metastases: (A, B) before nab‐paclitaxel; (C, D) after 2 cycles of nab‐paclitaxel; (E, F) after eight cycles of nab‐paclitaxel; (G, H) after two cycles of olaparib; (I, J) after four cycles of olaparib.

In July 2021, a chest CT examination revealed multiple nodules in both lungs, suggestive of metastasis. Next‐generation sequencing (NGS) performed on a peripheral blood sample using an Illumina platform did not detect any pathogenic or likely pathogenic variants. From August 2021 to February 2022, the patient underwent eight cycles of nab‐paclitaxel chemotherapy (nab‐paclitaxel 200 mg intravenously on Days 1 and 8, q3w). During the treatment, the lung metastases exhibited shrinkage, and the treatment response remained stable. However, after completing eight cycles, the patient developed severe hand‐foot syndrome, necessitating a pause in chemotherapy. Furthermore, CT examination indicated a tendency of enlargement in the lung metastatic lesions (Figure 2).

## 3 METHODS (DIFFERENTIAL DIAGNOSIS, INVESTIGATIONS, AND TREATMENT)

**FIGURE 3:**
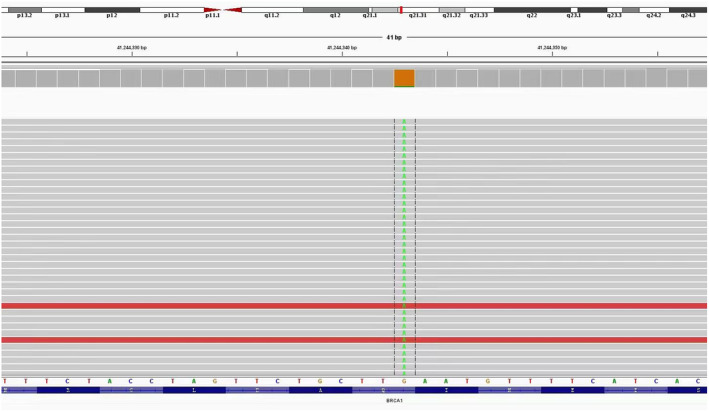
Screenshot of integrated genome viewer showing the sequencing reads for BRCA1.

**FIGURE 4:**
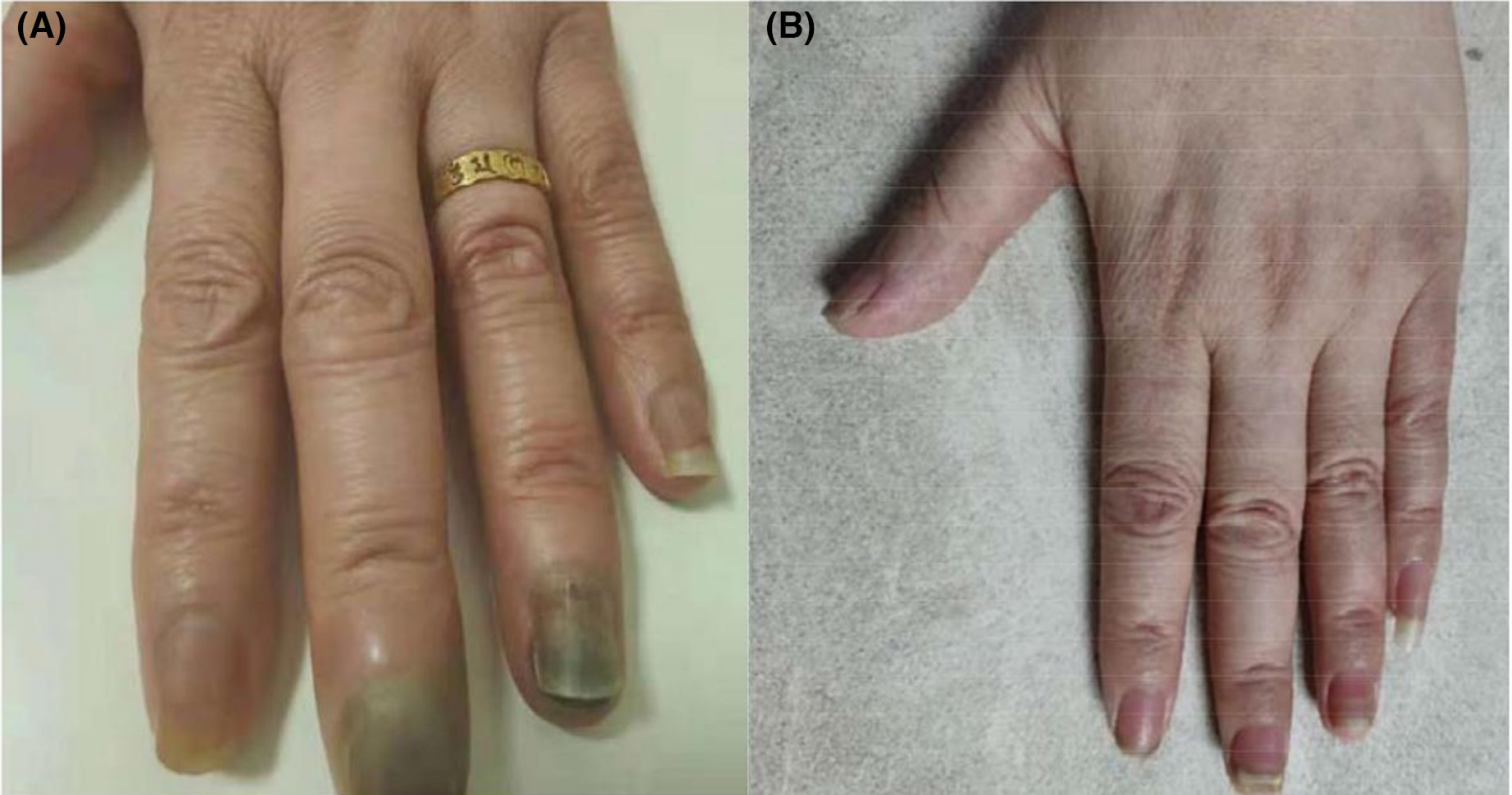
Hand‐foot syndrome: (A) After 8 cycles of nab‐paclitaxel; (B) After 4 cycles of Olaparib.

In order to identify a follow‐up treatment, the patient underwent peripheral blood NGS testing once again. It was pleasantly surprising that the NGS testing revealed the presence of the emerging BRCA1 gene p.Q1069* exon 11 mutation. This mutation might increase sensitivity to olaparib, a targeted drug specifically designed to address BRCA mutations (Figure 3). Since March 2022, the patient has taken olaparib 300 mg bid for 4 months. As expected, the metastases in the lungs shrank again after the patient received the treatment. She experienced a very good therapeutic effect with fewer side effects and good tolerance (Figure 4).

## 4 CONCLUSION AND RESULTS (OUTCOME AND FOLLOW‐UP)

This case gives some insights to clinicians: (1) BRCA gene mutations are not set in stone, treatment (especially chemotherapy) may alter the BRCA gene mutation status from peripheral blood genetic testing in breast cancer patients; (2) NGS testing leads to breast cancer treatment to be more precise, and dynamic monitoring of NGS testing is of great significance for follow‐up treatment in breast cancer.

## 5 DISCUSSION

BRCA mutation has long been considered as a breast cancer oncogenic factor highly related to familial inheritance.^3^, ^4^, ^5^ Hence, the predominant focus of most researchers lies in examining familial inheritance concerning BRCA mutations. There are currently no guidelines on how patients with advanced breast cancer should be continuously monitored for changes in the genetic phenotype. In this instance, we present a case wherein the patient's BRCA mutation deviated from the conventional understanding of being a germline mutation but rather emerged as an acquired gene mutation associated with chemotherapy. Our case report details the identification of a 7.2% plasma abundance of BRCA1 mutation in circulating tumor DNA in the patient's plasma. The patient's 41‐bp BRCA1 gene, p.q1069*, exhibited a nonsense mutation in exon 11, resulting in the alteration of the 3205 base from C to T and the 1069 amino acid from glutamine to terminator, consequently prematurely terminating the synthesis of the peptide chain. This mutation was documented in the Clinvar and UTAH databases as a pathogenic mutation. Notably, the initial peripheral blood NGS analysis of the patient revealed no discernible pathogenicity or identification of potential pathogenicity variants. However, subsequent to 8 cycles of NAB‐Paclitaxel treatment, a second peripheral blood NGS test uncovered a series of gene mutations, predominantly BRCA1 mutations. The patient subsequently experienced benefits from olaparib treatment, leading to a reduction in lung metastases.

Based on these facts, we infer that treatment with paclitaxel in this patient was responsible for the induction of the BRCA1 mutation, which mediated clinical resistance to paclitaxel and thus tumor progression. To support this hypothesis, we found strong evidence from numerous preclinical and clinical studies. Studies have confirmed that low concentrations of taxane‐based drugs can upregulate the expression of BRCA1 in normal breast cells and breast cancer cells with low BRCA1 expression.^6^, ^7^ In a portion of clinical cases of taxane resistance, various pathways of acquired taxane resistance were activated, in which BRCA1 expression was found to be up‐regulated.^8^ Regarding the mechanism of BRCA1 mutation leading to drug resistance of paclitaxel, we believe that it is related to homologous recombination (HR) pathway, which is a DNA repair mechanism in which BRCA proteins play a major role. At the DNA replication stage, defects in the HR pathway make tumors sensitive to anticancer drugs that induce double‐strand break, and HR is the dominant double‐strand break repair mechanism.^9^ BRCA1 also activates the mitotic spindle examination site, triggering apoptosis in response to microtubule injury. Nab‐paclitaxel inhibits mitosis of tumor cells by acting on tubulin. Therefore, mitotoxins, such as taxane, cannot exert their anticancer effects in tumors with BRCA1 mutations.^10^ Chabalier et al.^11^ reduced BRCA1 protein levels in MCF7 breast cancer cells, leading to paclitaxel‐based drug resistance through early inactivation of spindle checkup sites. Sung et al.^12^ found that by improving microtubule dynamics, stable microtubule formation of caspase‐8 accumulation in paclitaxel‐induced apoptosis was prevented. Further studies suggest that BRCA1 may be an important mediator of the stress response‐dependent C‐Jun N‐terminal kinase/stress‐activated protein kinase (JNK/SAPK) or p38/ mitogen‐activated protein kinase (P38 /MAPK) pathways of paclitaxel drugs.^13^ Taken together, these studies provide evidence that BRCA1 mutations lead to increased microtubule dynamics and impaired cell cycle checkpoints and signaling pathways, thereby reducing sensitivity to paclitaxel‐induced apoptosis. Therefore, we speculated that the patient developed a series of genetic mutations, mainly BRCA1 mutations, after receiving eight cycles of nab‐paclitaxel treatment.

In addition to the BRCA1 mutation, our patient was also detected with a 4.9% TP53 gene mutation. The patient had A missense mutation in the seventh exon of TP53 gene P.I255N, and the 764th base changed from T to A, resulting in the 255th amino acid changed from isoleucine to asparagine. TP53 gene is a kind of classical tumor suppressor gene, and its P53 protein is an important regulator of cell growth, proliferation, and damage repair.^14^, ^15^ Studies found that 22 tumor suppressor genes, including BRCA1, TP53, PTEN, APC, and CDKN1A, were directly and indirectly related to paclitaxel resistance.^16^ This suggests that paclitaxel selection pressure‐mediated BRCA mutations may induce a whole phenotype associated with acquired drug resistance, including the BRCA1 mutation, that tends to paclitaxel resistance. This underscores oncologists' awareness that antitumor treatments for patients may, in themselves, contribute to alterations in the tumor genetic phenotype, thereby resulting in tumor progression. Consequently, continual genetic sequencing proves advantageous in identifying such dynamic changes, guiding more precise targeted therapies.

In conclusion, our report provides clinical evidence that a nonexistent BRCA1 mutation detected in plasma circulating tumor DNA after disease progression is associated with disease progression and acquired resistance after treatment with nab‐paclitaxel. We believe that patients with advanced breast cancer often need to change their chemotherapy regimen because of disease progressed repeatedly. Under the selective pressure of different chemotherapy, the gene phenotype is in a state of dynamic change, which is highly correlated with the occurrence of acquired drug resistance and disease progression. Our report highlights the importance of the inaccuracy of one‐time genetic tests in assessing dynamic changes in BRCA1 mutations, and the importance of dynamic monitoring of NGS tests for follow‐up treatment of breast cancer. We suggest long‐term and regular dynamic evaluation of high‐risk patients who change their chemotherapy regimen, so as to provide more accurate and personalized treatment strategies for patients.

## AUTHOR CONTRIBUTIONS

**Xia‐Bo Shen:** Conceptualization; resources; software; validation; writing – review and editing. **Jia‐yi Wu:** Data curation; methodology; writing – original draft; writing – review and editing. **Jia‐ying Li:** Formal analysis; visualization. **Xi‐Ying Shao:** Formal analysis; project administration; resources; writing – review and editing. **Xiao‐Jia Wang:** Supervision; validation; writing – review and editing.

## FUNDING INFORMATION

This research received no external funding.

## CONFLICT OF INTEREST STATEMENT

The authors declare that the research was conducted in the absence of any commercial or financial relationships that could be construed as a potential conflict of interest.

## ETHICS STATEMENT

Ethics review and approval were not required for the study on human participants in accordance with the local legislation and institutional requirements. The patients/participants provided their written informed consent to participate in this study. Written informed consent was obtained from the individual(s) for the publication of any potentially identifiable images or data included in this article.

## Data Availability

The original contributions presented in the study are included in the article/supplementary material. Further inquiries can be directed to the corresponding author.
